# Navigating Nasal Surges: Understanding Catamenial Epistaxis

**DOI:** 10.7759/cureus.45767

**Published:** 2023-09-22

**Authors:** Nitish Batra, Rinkle Gemnani, Aditi Singh Thakur, Sunil Kumar, Sourya Acharya

**Affiliations:** 1 Department of Medicine, Jawaharlal Nehru Medical College, Datta Meghe Institute of Higher Education and Research, Wardha, IND; 2 Department of Obstetrics and Gynecology, Jawaharlal Nehru Medical College, Datta Meghe Institute of Higher Education and Research, Wardha, IND

**Keywords:** case report, endometrium, menstruation, epistaxis, catamenial

## Abstract

Catamenial epistaxis is a rare form of epistaxis (nosebleed) that occurs in women during menstruation due to hormonal changes. There are numerous hypotheses on the cellular mechanisms and pathophysiology of endometriosis. Endometriosis may present a wide range of symptoms depending on where endometrial tissue was implanted. This entity's diagnosis is neither simple nor difficult. There are numerous clinical and laboratory diagnostic techniques in use, but none of them is considered to be the best. Every woman who experiences recurrent symptoms (such as epistaxis and hemoptysis) of extrapelvic organs should be clinically suspicious of endometriosis because of its multipotent location and the variety of clinical manifestations of the condition. This case report demonstrates that periodic epistaxis may infrequently be the root cause of the extra pelvic endometrium in the nasal septum in a woman who has had treatment for recurrent pelvic discomfort and dysmenorrhea.

## Introduction

Endometriosis is the presence of endometrial glands and stroma outside the uterine cavity in up to 50% of infertile women and 15% of fertile women. It frequently happens in the pelvic organs, particularly in the ovary. Extragenital or extra pelvic endometriosis is the term used to describe occurrence outside the pelvis. Extra pelvic endometriosis has been described in almost every organ except the spleen, and it is difficult to diagnose and has high morbidity. Endometriosis is a pervasive disorder, but due to its highly diverse presentation, especially in extragenital endometriosis, it is still challenging to identify, diagnose, and treat [1]. There are several theories attempting to explain the pathogenesis, but none of them can explain all the localizations of endometriosis. There are retrograde menstruation and cell, lymphatic or hematogenous spread of endometrial cells, and others such as metaplasia, immunologic, and embryonic theories that can also explain extrapelvic endometrial implantation from different pains. However, ectopic endometrial implantation by retrograde menses is the most frequently accepted explanation for the etiology of endometriosis. Similarly, the migration of endometrial cells through the lymphatic and blood arteries can account for endometriosis in remote body areas. Endometriosis is a special kind of benign proliferation and metastasis as a result [2,3]. An exceptional site for endometriosis is the nasal mucosa. The presence of perimenstrual pain and epistaxis should increase the suspicion of nasal endometriosis in a woman of reproductive age [3]. We describe a case in which a woman experienced nasal tumefaction and epistaxis that had been cyclically recurring. The nasal nodule was surgically removed, confirming the endometriosis diagnosis. Other localizations were discovered through local and broad evaluations. Results have been good in the short- and medium-term. Despite the rarity of extra-pelvic localizations, endometriosis is a common disorder, having an unusual location in the nose.

## Case presentation

A 28-year-old woman presented to the emergency department with complaints of recurrent nosebleeds that occurred every month during her menstrual cycle. She reported having a history of heavy menstrual bleeding and dysmenorrhea for the past two years. The patient denied any history of trauma or nasal surgery. Upon general examination, she had a blood pressure of 100/50 mmHg and a heart rate of 128 bpm. Her respiratory rate was 23, and her oxygen saturation was 98% on room air. There was pallor, no lymphadenopathy, no hepatomegaly, and moderate splenomegaly. Systemic examination was unremarkable. Blood investigations revealed anemia and thrombocytopenia. All the laboratory parameters are shown in Table 1.

**Table 1:** Laboratory parameters of the patient with reference range PT-INR: prothrombin time and international normalized ratio, TSH: thyroid stimulating hormone

Laboratory investigations	Value	Biological reference range
Haemoglobin	8.3	13-15 g/dl
Mean corpuscular volume	73.8	79-100 fL
Total leukocyte count	4,200	4000-11,000/cumm
Platelet count	1,00,000	1,50,000–4,50,000/cumm
PT-INR	1.2	<=1.1
TSH	2.36	0.465-4.68
Serum urea	68	5-20 mg/dl
Serum creatinine	1.1	0.3-1.2 mg/dl
Serum sodium	138	135-145 mmol/L
Serum potassium	4.0	3.5-5.5 mmol/L
Serum albumin	4.0	3.5-5.5 g/dl

**Figure 1:**
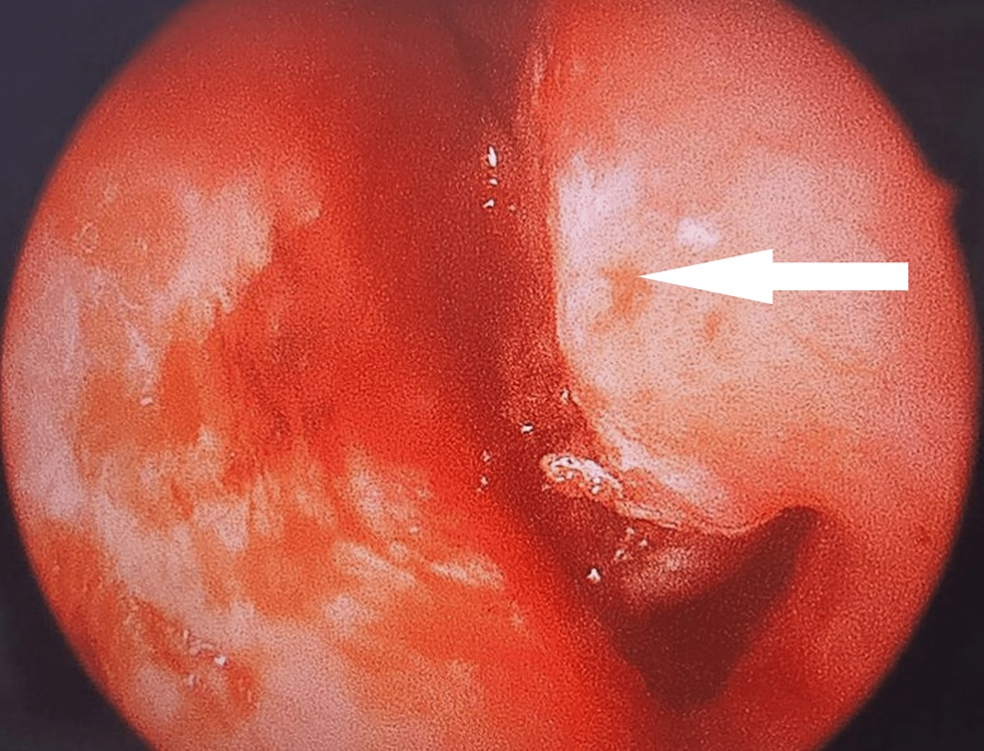
Nasal endoscopy showing granulation tissue

**Figure 2:**
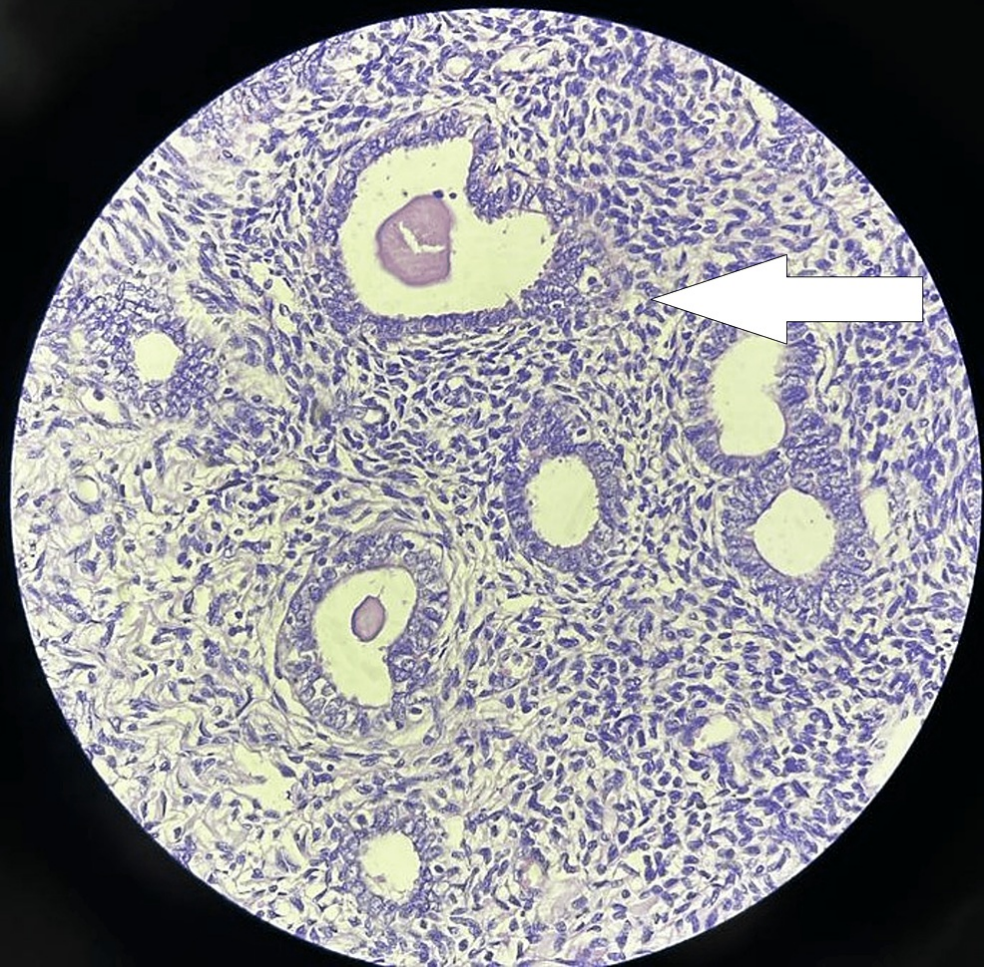
Nasal granulation tissue biopsy suggestive of endometrial tissue in a proliferative state

Paranasal CT and facial MRI were performed to rule out malignancy and other pathological conditions. An otolaryngologist's opinion was taken, and a nasal endoscopy was done, which was suggestive of granulation tissue (Figure 1). The granulation tissue was excised, and the sample was sent for tissue analysis which revealed endometrial tissue in a proliferative state (Figure 2).

The patient was diagnosed with catamenial epistaxis and was treated with intranasal packing with gauze soaked in a solution of epinephrine and lidocaine and was commenced on hormonal therapy. She was advised to avoid physical exertion during her menstrual cycle and take over-the-counter non-steroidal anti-inflammatory drugs for pain relief. The patient was followed up in the outpatient department after one month. She reported no further episodes of epistaxis since her initial presentation. Her menstrual cycle had become regular with the use of oral contraceptive pills, and the dysmenorrhea had resolved. A repeat nasal examination showed no evidence of any active bleeding or nasal abnormalities.

## Discussion

Endometriosis is a prevalent gynecological condition affecting 10-15% of women of reproductive age. Numerous theories have been proposed to explain the pathophysiology, but none of them can account for all the localizations of endometriosis [1]. Retrograde menstruation, lymphatic or hematogenous dissemination of endometrial cells, metaplasia, and immune and embryologic theories, among others, can also account for extra pelvic endometrial implantation resulting from various symptoms [1]. Extra pelvic endometriosis often occurs within the abdominal wall, with surgical interventions on the uterus, particularly cesarean sections, being a common trigger. Women affected by abdominal wall endometriosis typically experience persistent, localized abdominal pain unrelated to their menstrual cycle. This discomfort is frequently unconventional, leading to potential misdiagnosis, and may also be accompanied by the detection of a palpable lump in the surgical scar region [2].

Thoracic endometriosis is a medical condition characterized by the presence of abnormal endometrial tissue outside the uterus, specifically in the pleura, pericardium, and occasionally the diaphragm. This often presents as catamenial hemothorax, which is the primary manifestation of a broader syndrome called thoracic endometriosis syndrome. This syndrome encompasses four other conditions: catamenial hemothorax, catamenial hemoptysis, endometriotic lung nodules, and catamenial chest pain. These symptoms share a common trait: their occurrence is linked to the menstrual cycle [1-3]. Extra pelvic endometriosis can also manifest within the liver and gallbladder, though such occurrences are exceedingly rare.

Limited international literature documents around 14 cases of liver endometriosis, predominantly characterized by patients experiencing discomfort and a sense of heaviness in the upper right abdominal quadrant. In some instances, liver endometriosis has presented with symptoms akin to obstructive jaundice [4]. The occurrence of gallbladder endometriosis is exceptionally uncommon, with merely two reported cases in the available literature. Additionally, certain women encounter endometriosis within their gastrointestinal tract, a condition termed intestinal endometriosis. Notable symptoms of bowel endometriosis encompass rectal bleeding and pain, distressing bowel movements, reduced appetite, cramp-like abdominal pains, nausea, vomiting, constipation, and diarrhea, as well as abdominal bloating and increased abdominal gas. These symptoms intensify during menstruation [3-4]. The primary locations for extra pelvic intestinal endometriosis are typically found at the terminal part of the ileum (the final segment of the small intestine), the cecum (the initial part of the large intestine), and the appendix [4-5]. In addition to the more uncommon occurrences of endometriosis, noteworthy instances involve its presence within larger muscles like the adductor compartment, the rectus abdominal muscle, and the gluteal muscle. The latter can lead to catamenial sciatica.

A solitary case report within the literature recounts nerve endometriosis, precisely detailing endometriosis affecting the L5 nerve, resulting in gluteal atrophy and sciatica [5-6]. Other uncommon causes of epistaxis, like mitral stenosis as described by Kumar et al., should also be excluded [7]. Although extra pelvic endonasal endometriosis is uncommon, perimenstrual discomfort and epistaxis in a woman of reproductive age may raise suspicions of nasal endometriosis. Treatment may include hormonal therapy, non-steroidal anti-inflammatory drugs, and intranasal packing, depending on the severity of the condition. As far as surgical treatment and use of oral contraceptive pills in the treatment and prevention of extra pelvic endometriosis is concerned, there is no data available in the literature search [8].

## Conclusions

Catamenial epistaxis is a rare but significant cause of recurrent epistaxis in women during their menstrual cycle. Identifying this condition early and providing appropriate treatment to prevent further complications is essential. Consultation with an obstetrician-gynecologist is recommended to evaluate and manage any menstrual abnormalities that may be associated with this condition. Depending on the place of endometrial tissue implantation, endometriosis can be expressed with a wide variety of symptoms. The diagnosis of this entity is neither easy nor routine. Numerous clinical and laboratory diagnostic techniques have been used, but none of them is the gold standard. The multipotent localization of endometriosis in combination with the wide range of its clinical expression should raise clinical suspicion in every woman with periodic symptoms of extra pelvic organs.
